# Soft X-ray nanospectroscopy for quantification of X-ray linear dichroism on powders

**DOI:** 10.1107/S1600577521004021

**Published:** 2021-05-19

**Authors:** Selwin Hageraats, Mathieu Thoury, Stefan Stanescu, Katrien Keune

**Affiliations:** a Conservation and Science, Rijksmuseum, PO Box 74888, 1070DN Amsterdam, The Netherlands; b IPANEMA, CNRS, Ministère de la Culture et de la Communication, Université de Versailles Saint-Quentin-en-Yvelines, USR 3461, Université Paris-Saclay, 91128 Gif-sur-Yvette, France; c Van ’t Hoff Institute for Molecular Science, University of Amsterdam, PO Box 94157, 1090 GD Amsterdam, The Netherlands; d Synchrotron SOLEIL, 91192 Gif-Sur-Yvette Cedex, France

**Keywords:** XLD, STXM, soft X-rays, ZnO

## Abstract

X-ray linear dichroism (XLD) is a fundamental property of many ordered materials that can for instance provide information on the origin of magnetic properties and the existence of differently ordered domains. Conventionally, measurements of XLD are performed on single crystals, crystalline thin films, or highly ordered nanostructure arrays. Here, it is demonstrated how quantitative measurements of XLD can be performed on powders, relying on the random orientation of many particles instead of the controlled orientation of a single ordered structure. The technique is based on a scanning X-ray transmission microscope operated in the soft X-ray regime. The use of a Fresnel zone plate allows X-ray absorption features to be probed at ∼40 nm lateral resolution – a scale small enough to probe the individual crystallites in most powders. Quantitative XLD parameters were then retrieved by determining the intensity distributions of certain diagnostic dichroic absorption features, estimating the angle between their transition dipole moments, and fitting the distributions with four-parameter dichroic models. Analysis of several differently produced ZnO powders shows that the experimentally obtained distributions indeed follow the theoretical model for XLD. Making use of Monte Carlo simulations to estimate uncertainties in the calculated dichroic model parameters, it was established that longer X-ray exposure times lead to a decrease in the amplitude of the XLD effect of ZnO.

## Abstract 2

It is shown how soft X-ray nanospectroscopy maps of powders (particle size ∼200 nm) can be analyzed to quantitatively retrieve X-ray linear dichroism (XLD) parameters. A computational modeling procedure is described that can be used in conjunction with Monte Carlo simulations to prove statistical dissimilarity of XLD parameters between different samples.

## 1. Introduction   

The X-ray absorption behavior of materials at and above elemental absorption edges can provide a wealth of information on oxidation states, coordination symmetry, and distortions in the coordination environment. Depending on the type of information that is required, one may either probe the discrete bound-to-bound transitions directly around the absorption edge (X-ray absorption near-edge structure, abbreviated XANES) or the bound-to-continuum oscillations further above the absorption edge (extended X-ray absorption fine structure, abbreviated EXAFS). When studying ordered materials, such as crystals or certain polymers, X-ray absorption spectra recorded with linearly polarized X-rays may exhibit a distinct angle-dependency. That is, the intensities of some absorption features fluctuate upon rotating the ordered structures relative to the axis of polarization. This effect is called X-ray linear dichroism (XLD) and can either be related to anisotropies in the electronic or the magnetic properties of materials. This first property is called X-ray natural linear dichroism (XNLD) and occurs due to charge anisotropies in certain ordered materials. Such ordered materials can either be crystalline, in which case they have a non-cubic crystal structure with a rotation axis of order three or higher (Brouder, 1990 ▸), or non-crystalline, in which case the material has for instance formed non-covalent ordered structures of low symmetry (*e.g.* molecular adsorbates) (Zharnikov & Neuber, 2000 ▸; Fu & Urquhart, 2005 ▸). The second property is called X-ray magnetic linear dichroism (XMLD) and occurs in crystals that possess a preferential magnetic axis such as in ferromagnetic and antiferromagnetic materials (Thole *et al.*, 1985 ▸; Schwickert *et al.*, 1998 ▸; Kortright & Kim, 2000 ▸; Kuiper *et al.*, 1993 ▸; Spanke *et al.*, 1998 ▸; Antel Jr *et al.*, 1999 ▸; Scholl *et al.*, 2000 ▸). The study of these two properties can provide information on the configuration of impurities in host lattices (Gaudry *et al.*, 2005 ▸; Schauries *et al.*, 2013 ▸), the magnetic characteristics of materials (Spanke *et al.*, 1998 ▸; Antel Jr *et al.*, 1999 ▸; Scholl *et al.*, 2000 ▸) and the existence of order in organic systems (Zharnikov & Neuber, 2000 ▸; Smith & Ade, 1996 ▸).

Experimentally, XLD is typically studied by rotating a sample with respect to the electric field vector of the polarized X-rays or vice versa. For each angle, an X-ray absorption spectrum or the absorption at a few characteristic features is recorded. For this set-up to work, it is crucial that within the measured sample volume all material has the same spatial order. This typically requires either a single crystal (Gaudry *et al.*, 2005 ▸; Juhin *et al.*, 2008 ▸), a crystalline thin film (Spanke *et al.*, 1998 ▸; Scholl *et al.*, 2000 ▸), a highly ordered array of nanostructures (Chiou *et al.*, 2004 ▸) or an ordered ensemble of molecular adsorbates (Zharnikov & Neuber, 2000 ▸).

Alternatively, in macroscopically disordered materials, the spatial distribution of differently oriented domains can be spatially resolved using scanning transmission X-ray microscopy (STXM) or X-ray photoelectron emission microscopy (XPEEM) in combination with linearly polarized X-ray beams (Spanke *et al.*, 1998 ▸; Scholl *et al.*, 2000 ▸; Chiou *et al.*, 2004 ▸; Smith & Ade, 1996 ▸). In STXM, the X-ray beam is focused down to a spot size of the order of tens of nanometres and raster scanned across a sample. The transmitted X-rays are recorded per point and can be reconstructed to a sample image. XPEEM, on the other hand, makes use of the photoelectric effect with linearly polarized X-rays tuned to be in resonance with core electron binding energies. Using either a tightly focused beam (then called scanning X-ray photoelectron microscopy, abbreviated SPEM) or full-field irradiation, XLD images can be obtained with a spatial resolution in the order of tens of nanometres.

It is proposed here to take advantage of the extraordinarily high spatial resolution of STXM to quantify XLD properties on powders — hereby circumventing the need for single crystals or other highly ordered sample structures. The proposed technique makes use of the random orientation of many crystallites to obtain distributions whose width and skewness hold information on the XLD parameters. Recognizing the quadratic dependency of the absorption cross-section on the amplitude of the electromagnetic perturbation following Fermi’s golden rule (Stöhr, 1992 ▸), and the dependency of the perturbation on the projection of the polarization vector **e** onto the transition dipole moment, the following model for XLD is used,

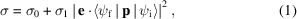

where σ is the absorption cross-section for a transition between initial state ψ_i_ and final state ψ_f_, **p** is the electron momentum operator, and σ_0_ and σ_1_ are the relative amplitudes of the angle-independent and angle-dependent fractions of the absorption cross-section. Writing ψ_f_|**p**|ψ_i_ as the transition dipole moment vector μ_if_, one obtains

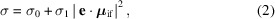

which can be expressed as a function of the angle δ between the polarization vector and transition dipole moment according to

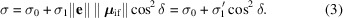

Transforming to computationally more straightforward polar coordinates and defining the polarization vector **e** to have coordinates 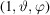 = 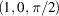 , one obtains

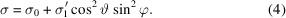

To demonstrate how XLD can be quantified on powders, a proof-of-principle study is described here on ZnO: a semiconductor that has been shown previously to exhibit XNLD based on Zn *K*-edge and O *K*-edge studies of ZnO single crystals and thin ZnO epilayers, respectively (Goulon *et al.*, 2007 ▸; Preston *et al.*, 2008 ▸). Here, XNLD is studied at the Zn *L*-edge (∼1030 eV) in order to facilitate compatibility with a soft X-ray STXM at the highest possible photon flux. Although this study quantifies XNLD, the mathematical similarity with XMLD means that such dichroic parameters could be similarly quantified using the proposed methodology.

This work is based in part on the experimental data presented first by Hageraats *et al.* (2020 ▸), and offers a revised interpretation of the results.

## 2. Methods   

### 2.1. Sample preparation   

Samples from four different batches of ZnO were analyzed. Three of these were zinc white pigments produced using the French (indirect) vapor oxidation process and were obtained from modern pigment manufacturers. The three zinc white samples are henceforth referred to as FrMod1-3. A fourth sample of ultrapure ZnO was lab-synthesized by combusting high-purity Zn-foil (99.99%, 0.125 mm thickness; Advent Research Materials Ltd) in a stainless steel airtight glovebox. A controlled atmosphere was created by outgassing and purging with argon three consecutive times and then introducing an 80/20 mixture of Ar/O_2_ (Air Liquide, Ar > 99.99% and O_2_ > 99.995% purity) into the glovebox up to a pressure of 1 bar. After combustion, the ultrapure ZnO powder was collected in the inert atmosphere on a glass plate. X-ray photoelectron spectroscopy (XPS) analysis of the lab-synthesized batch showed no detectable impurities. This sample is henceforth referred to as ZnO-L.

All four ZnO samples were prepared for STXM analysis by suspending a few hundred µg of ZnO in ∼1 ml of iso­propanol, sonicating the suspension for several minutes and then drop casting a ∼5 µL droplet onto a 100 nm thin SiN window heated to ∼100°C. After several seconds, all iso­propanol evaporated, leaving small amounts of ZnO dispersed over the window surface.

### 2.2. Scanning transmission X-ray microscopy   

STXM experiments were conducted at the HERMES beamline of the SOLEIL synchrotron. The undulator radiation was monochromated with a monochromator that supports transmission of photons up to 1600 eV. The STXM (Research Instruments GmbH) was equipped with a 30 nm outer ring width Fresnel zone plate (FZP) and 50 µm order-sorting aperture to focus the monochromated beam down to a diameter of approximately 40 nm. SiN windows supporting the various ZnO samples were fixed to an *XYZ* scanning stage, *Z* being the direction collinear with the X-ray beam propagation and used to bring the sample onto the FZP focal plane. STXM images were obtained by raster scanning the *XY* position and recording the transmitted photons using a photomultiplier tube (PMT). Based on low-resolution overview images of the SiN windows, the ZnO dispersions were found to consist of numerous clusters of several tens to hundreds mostly unstacked ZnO crystallites — each cluster measuring several µm in diameter. Therefore, the dimensions of the STXM images were set to be in the range 3 µm × 3 µm to 6 µm × 6 µm. XANES contrast maps were obtained on all four samples (FrMod1-3, ZnO-L) by recording ∼150 consecutive images at X-ray energies in the range 1015–1050 eV, thereby obtaining hyperspectral Zn *L*-edge maps. In addition, the FrMod1 and ZnO-L samples were analyzed by choosing just two diagnostic energies (1031 and 1033 eV) that show the strongest XNLD behavior, hereby minimizing the effect of possible radiation damage due to prolonged exposure to the high-flux X-ray beam and maximizing the number of spatial points that can be analyzed in a reasonable timeframe.

### 2.3. Data processing   

#### 2.3.1. Visualizing XNLD contrast   

**Figure 1:**
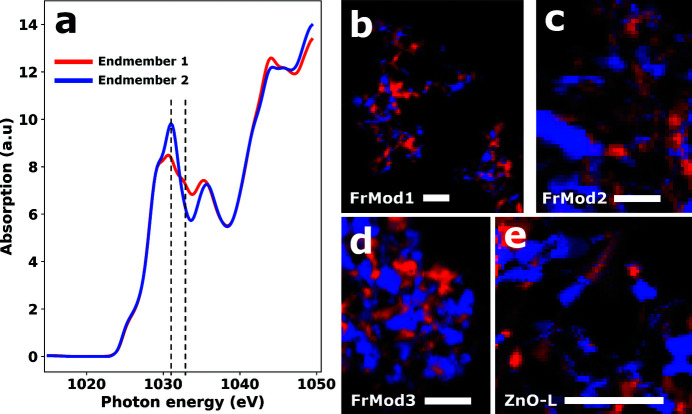
SiVM-NNLS analysis of the four hyperspectral STXM data sets. (*a*) Plot of endmembers 1 and 2. (*b*–*e*) False color representations of the NNLS fits with endmembers 1 (red channel) and 2 (blue channel) of samples FrMod1-3 and ZnO-L. All scale bars have a length of 1 µm.

STXM energy stacks first were aligned using a Fourier alignment algorithm and transformed from transmission to optical density (OD) in the *aXis2000* software. XNLD contrast was visualized in the hyperspectral STXM-XANES maps by running the simplex volume maximization (SiVM) endmember selection algorithm (adapted from the *DataHandlerP* software) (Thurau *et al.*, 2012 ▸) and fitting the data with non-negative least-squares (NNLS) using the first four endmembers as input factors. Following the SiVM approach, endmember spectra are individual spectra from the dataset which, in the most ideal case, are thought to constitute the *purest* representations of the different compounds present in the sample. In this ideal case, the endmembers of a pure and dichroic sample can be interpreted as those spectra exhibiting the largest dichroic differences. Following the initial SiVM endmember selection, the endmembers were updated by averaging all spectra scoring primarily high on endmembers 1 and 4, chosen based on their low degree of covariance. The resulting endmembers [see Fig. 1 ▸(*a*)] were found to represent two distinctly different **e** · **μ**_if_ projections. A second NNLS fit with the two updated endmembers then yielded weight matrices that were used as red and blue channels to produce false color R(G)B images showing the XNLD contrast among individual ZnO crystallites in the four hyperspectral Zn *L*-edge STXM datasets.

#### 2.3.2. Quantifying XNLD   

**Figure 2:**
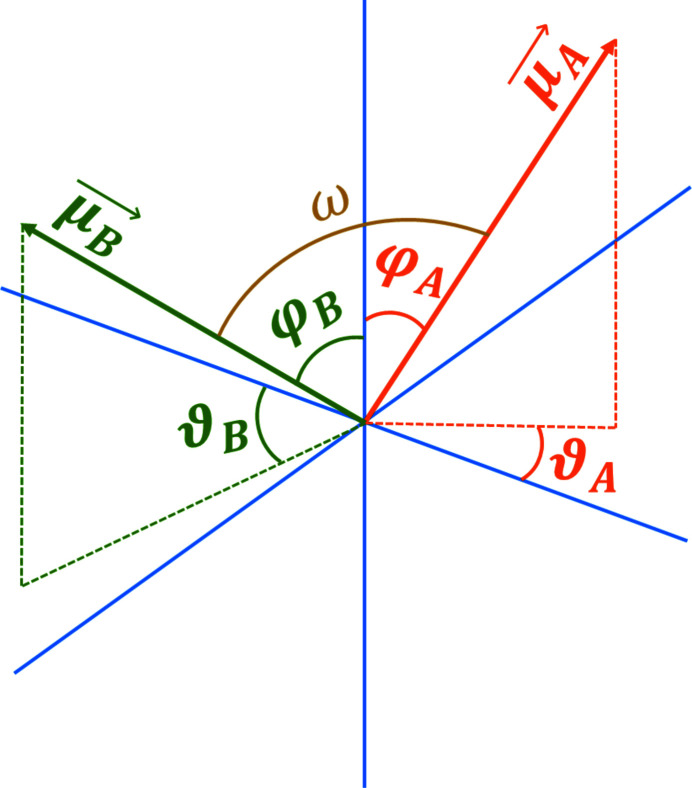
Illustration of the angular coordinates of transition dipole moments 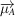 and 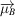 , corresponding to the transitions at the two diagnostic energies.

From the two dichroic spectral endmembers, two diagnostic energies were determined. These absorption features were found to respond most strongly — and oppositely — as a function of the projection angle δ, making them suitable for quantification of the XNLD parameters. As the authors discussed in a previous paper on soft X-ray nanospectroscopy of ZnO, these two diagnostic energies are thought to correspond to transitions between Zn 2*p* (*J* = 3/2) and Zn 4*d* derived states (Hageraats *et al.*, 2020 ▸). The anisotropic character of the Zn 4*d* derived states causes both these transitions to have a dichroic character. Furthermore, the different symmetries and/or angular offset among different Zn 4*d* derived states mean that the XLD model proposed in equation (4) needs to be extended to include a phase shift ω. This angle ω represents the angle between the transition dipole moments for these two diagnostic transitions. The complete model for two symmetrically distinct transitions *A* and *B* then becomes

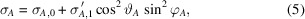




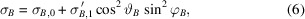

with

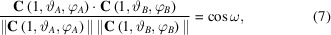

where the operator **C** indicates transformation of the vector from spherical coordinates to Cartesian coordinates. Fig. 2 ▸ illustrates the relation between the two transition dipole moments 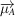 and 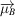 , with all angles indicated.

In order to link the model to the experimental data, all spectra were normalized to their integral. This corrects for the local OD and yields values that are approximately proportional to the absorption cross-section. Since relative σ values provide sufficient information to quantitatively compare the XNLD behavior of different powders, the proportionality factor is here assumed to be unity, simply giving σ_*A*_ = *I*_*A*_ and σ_*B*_ = *I*_*B*_, where *I*_*A*_ and *I*_*B*_ are the experimentally measured and normalized intensities at the two diagnostic energies 1031 and 1033 eV, respectively. Assuming no significant overlap between the two absorption features, the model for the experimental data then becomes

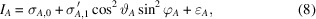




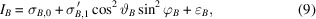

with the relation between ϑ_*A*_, φ_*A*_, ϑ_*B*_, φ_*B*_ still dictated according to equation (7). Here, 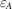 and 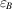 are experimental errors that are here both assumed to be normally distributed around zero with an expected absolute value |ɛ|. Experimental distributions were determined by binning all values of *I*_*A*_ and *I*_*B*_ from a certain dataset in 30 bins.

**Figure 3:**
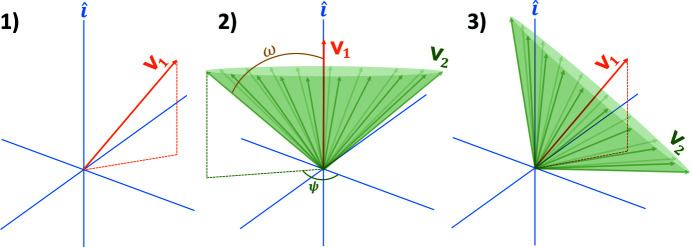
Illustration of the procedure for generating random vector pairs **v**_1_/**v**_2_. In step 1, a random vector is **v**_1_ is picked from a uniform distribution on a unit sphere. In step 2, **v**_1_ is temporarily defined along 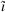 , around which a cone is defined with projection 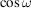 . From this cone, **v**_2_ can be picked by choosing a value of ψ from a uniform distribution [0, 2π]. In step 3, both **v**_1_ and **v**_2_ are rotated so that **v**_1_ is back to its original position.

Model distributions were determined by picking approximately 10^5^ pairs of random unit vectors that represent the two transitions dipole moments of ∼10^5^ individual crystallites. According to a procedure laid out in Fig. 3 ▸, each first vector of the random vector pairs (**v**_1_) was determined by randomly picking *x*, *y*, and *z* coordinates from a single Gaussian distribution and normalizing its length to unity, yielding vectors whose coordinates are uniformly distributed on a unit sphere (Muller, 1959 ▸). Each second vector (**v**_2_) is picked by recognizing that all vectors under an angle ω with respect to the first vector exist on a cone with projection 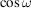 and an azimuthal angle ψ. Therefore, each second vector can be picked by randomly sampling an angle ψ from a uniform distribution [0, 2π] — yielding polar coordinates (1, ψ, ω) — determining its Cartesian coordinates in a coordinate system with **v**_1_ pointing along 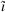 , and rotating the vector using Rodrigues’ rotation formula,

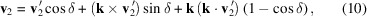

where 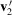 = 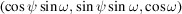 is the randomly picked second vector of the vector pair prior to rotation, 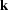 = 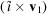 is the axis of rotation, and 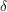 = 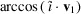 is the rotation angle.

These sets of randomly oriented unit vectors can then be plugged into equations (8) and (9) to obtain model distributions of *I*_*A*_ and *I*_*B*_ for given dichroic model parameters σ_*A*,0_, 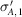 σ_*B*,0_, and 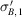 and expected experimental error |ɛ|. Quantification of the dichroic model parameters and experimental error for given experimental distributions can then be performed by applying a fitting algorithm.

Central to the fitting algorithm is a cost function *F*_C_ that measures the goodness of fit of a model distribution [equations (8) or (9)] with respect to an experimental distribution. *F*_C_ takes as input a set of dichroic model parameters ( 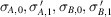 ), an expected experimental error (|ɛ|), and a pre-calculated set of random unity vectors. It then calculates a model distribution, compares it with the experimental distribution, and returns the sum of squared errors. Optimization of the model parameters and estimation of the expected experimental error was done by means of a tailored gradient descent method. The gradient descent method works by calculating the gradient of the cost function *F*_C_ around an estimate 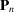 = 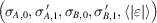 and updating the estimation according to

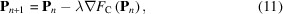

where λ is a constant that is manually chosen so as to ensure fast and reliable convergence onto local minima. The parameter vector **P**_*n*_ that produces a global minimum of the cost function *F*_C_ is regarded as the best approximation of the experimental distribution.

To facilitate the estimation of a realistic uncertainty in the calculated dichroic model parameters, a Monte Carlo simulation of synthetic datasets was performed for each experimental distribution. Synthetic datasets of experimental distributions *I*_*A*_ and *I*_*B*_ were calculated by assuming that the estimates of the dichroic model parameters and experimental error — obtained through the fitting algorithm — are close to the true values. Accurate synthetic copies of the experimental distributions can then be obtained by picking a number of vector pairs that is equal to the number of measurements of *I*_*A*_ and *I*_*B*_, with a number of unique orientations equal to the number of crystallites captured in the experimental dataset. Each vector pair is then evaluated according to equations (8) and (9), yielding two synthetic distributions. Each synthetic dataset was modeled using the previously described fitting algorithm, yielding multiple estimates of each dichroic model parameter. The standard deviation among these estimates was taken as *u*_*P*,exp_: the uncertainty in an estimate of model parameter *P* due to the limited number of measurements that constitute the experimental datasets.

## 3. Results   

To give an idea of the extent and the nature of the XNLD of ZnO powders, Figs. 1 ▸(*b*)–1(*e*) shows false-color XNLD images of the samples FrMod1-3 and ZnO-L determined from NNLS fits of the hyperspectral STXM datasets with the endmembers shown in Fig. 1 ▸(*a*). Distributions of ZnO crystallites can be seen that exhibit roughly 50% red and 50% blue particles, meaning their orientation with respect to the polarization axis is either most similar to that of endmember 1 or that of endmember 2. The ability to nearly binarily classify particles as either red or blue means that the two endmembers do not represent extremities in the **e** · **μ**_if_ projections but rather intermediaries. The true extent of the XNLD effect in ZnO therefore must exceed the effect that separates endmembers 1 and 2.

**Figure 4:**
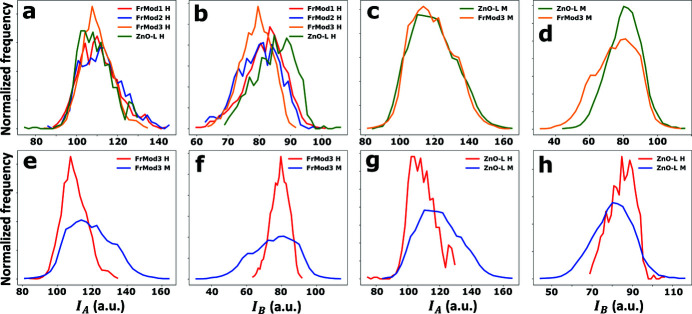
Experimental distributions of *I*_*A*_ and *I*_*B*_ for (*a*, *b*) the hyperspectral data sets, (*c*, *d*) the multispectral data sets, (*e*, *f*) the data recorded on the FrMod3 sample, and (*g*, *h*) the data recorded on the ZnO-L samples.

Fig. 4 ▸ shows the experimental distributions of *I*_*A*_ and *I*_*B*_, grouped together based on whether the data is obtained from a hyperspectral STXM energy stack [Figs. 4 ▸(*a*) and 4(*b*)], a two-energy STXM stack [Figs. 4 ▸(*c*) and 4(*d*)], only the FrMod3 sample [Figs. 4 ▸(*e*), and 4(*f*)], or only the ZnO-L sample [Figs. 4 ▸(*g*) and 4(*h*)]. From these distributions, it can clearly be surmised that the distributions obtained from hyperspectral STXM energy stacks are narrower than those obtained from just two energies. Furthermore, the *I*_*B*_ distribution obtained from a two-energy STXM stack recorded on the ZnO-L sample appears to deviate significantly from the same distribution obtained on the FrMod3 sample [Fig. 4(*d*) ▸].

In order to quantify these visually observed differences in terms of dichroic model parameters, it is first necessary to estimate the angle ω. Although this angle does partly dictate the relation between *I*_*A*_ and *I*_*B*_ [see equation (7)], random variations in φ_*A*_, φ_*B*_ and experimental noise weaken this relation to such an extent that it was found to be impossible to retrieve an accurate measure of ω through analysis of the raw data. Instead, an estimate was made based on the spectral endmembers shown in Fig. 1 ▸(*a*). Given these two distinctly different projections of **μ**_if_ onto **e**, there appears to be a negative correlation, corresponding to a value of ω = 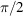 for a cos^2^ϑ relationship. In terms of the orbitals involved in the transitions related to the two diagnostic energies, this means that the orbitals of the two Zn 4*d* derived states (see Section 2.3.2) have a 90° angular offset.

**Figure 5:**
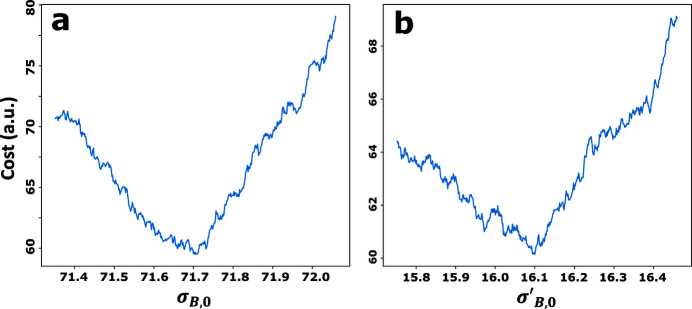
The cost of fitting an experimental distribution of *I*_*A*_ around a minimum, evaluated as a function of dichroic model parameters σ_*B*,0_ (*a*) and 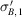 (*b*).

This value was plugged into the model proposed in equations (8) and (9) to define the cost function central to the fitting algorithm. Convergence of the algorithm onto global minima was found to be problematic due to the noisy nature of the cost function. This property can be observed in Fig. 5 ▸, which shows the cost in fitting an experimental distribution *I*_*A*_ as a function of dichroic parameters σ_*B*,0_ and 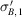 , evaluated around what is thought to be the global minimum. This noisy nature is most likely related to the fact that the model distributions are not continuous functions, but rather consist of bins that contain an inherently finite number of samples. Small changes in the model parameters may only shift a few samples to new bins, a process that is prone to introduce high-frequency shot noise to the cost function.

These noise characteristics were dealt with by introducing three modifications to the default gradient descent method. First, the parameter space over which local gradients were evaluated was set a lot bigger than usual, so as to take only the longer-range slopes into account and avoid sensitivity to noise. Second, as the cost function noise may still occasionally cause gross overestimation of the gradient and force the algorithm far away from the (local) minimum, the size of each optimization step was normalized to approximately 0.005σ_*A*,0_ for the first 20–50 iterations and approximately 0.001σ_*A*,0_ for all subsequent iterations. Third, due to the vast number of local minima, the algorithm was found to rarely converge on the same parameters twice for a given experimental or synthetic distribution. For this reason, after each convergence an initial guess was reinitialized by randomly mutating the dichroic parameters corresponding to the lowest previously found value of the cost function. Per 1000 iterations, the algorithm converged 5–15 times with a cost at most 25% higher than the absolute lowest cost value found. As a 25% difference in cost is not thought to be significant for data with noise levels this high, all of these convergences could be used together to estimate the dichroic parameters and determine *u*_*P*,mod_: the uncertainty in an estimate of model parameter *P* due to the modeling error.

**Figure 6:**
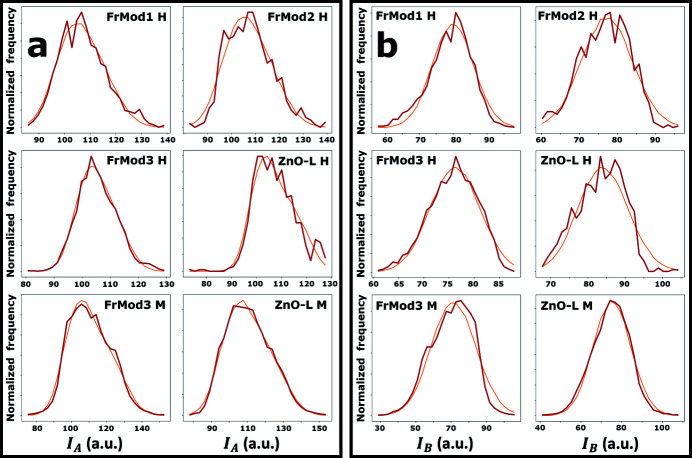
Experimental distributions (red) of *I*_*A*_ (*a*) and *I*_*B*_ (*b*) fitted according to the dichroic model postulated in equations (8) and (9) (orange).

Fig. 6 ▸ shows the fits obtained for each of the experimental distributions, taking the dichroic parameters obtained for ω = 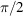 and iterating the optimization algorithm 10000 times. It can be seen that the width and skew of the experimental distributions is mostly modeled accurately in the case of *I*_*A*_, but less accurately in the case of *I*_*B*_. To be precise, the experimental distributions of *I*_*B*_ tend to have a negative skew, while the model only permits a positive skew.

**Figure 7:**
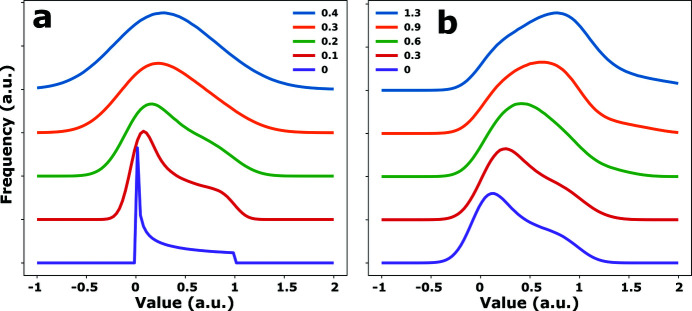
(*a*) PDFs of cos^2^ϑsin^2^φ + ɛ evaluated for values of ɛ ranging from zero (purple) to 0.4 (blue). (*b*) PDFs of 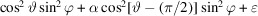 evaluated for ɛ = 0.15 and values of α ranging from 0 (purple) to 1.3 (blue).

This issue is illustrated in Fig. 7 ▸(*a*), where it shows the numerically approximated probability density function (PDF) of cos^2^ϑsin^2^φ + ɛ for randomly oriented unit vectors with different levels of noise ɛ. The PDF clearly has a positive skew and can only be made (nearly) symmetrical by adding a high level of noise. One way in which a PDF with the right (negative) skew can be obtained is to add an out-of-phase contribution. Fig. 7 ▸(*b*) shows the PDFs of cos^2^ϑsin^2^φ + αcos^2^ϑ′sin^2^φ′ + ɛ with every set of angles describing two perpendicular vectors: **C**(1, ϑ, φ) · **C**(1, ϑ′, φ′) = 0. Values of α were chosen between 0 and 1.3 and the noise level |ɛ| was fixed at 0.15. In Fig. 7 ▸(*b*) it can be seen that, when the in-phase and out-of-phase contributions become roughly equal, the skew becomes negative. With regard to the experimental distributions of *I*_*B*_, this means that the intensity of the feature at 1033 eV must have contributions from two different transitions with transition dipole moments oriented at a large angle relative to one another. Given the width of the absorption features (see Fig. 1 ▸), it is therefore likely that the absorption measured at 1033 eV has contributions both from the intense feature at 1031 eV and a transition with different symmetry with an energy around 1033 eV.

This means that the model proposed in equations (8) and (9) is incomplete in the sense that it does not consider the overlap between transitions with non-parallel transition dipole moments. The following alternative model is therefore proposed,

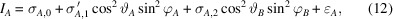




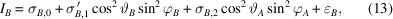

with the relation between ϑ_*A*_, φ_*A*_, ϑ_*B*_, φ_*B*_ still dictated according to equation (7).

**Figure 8:**
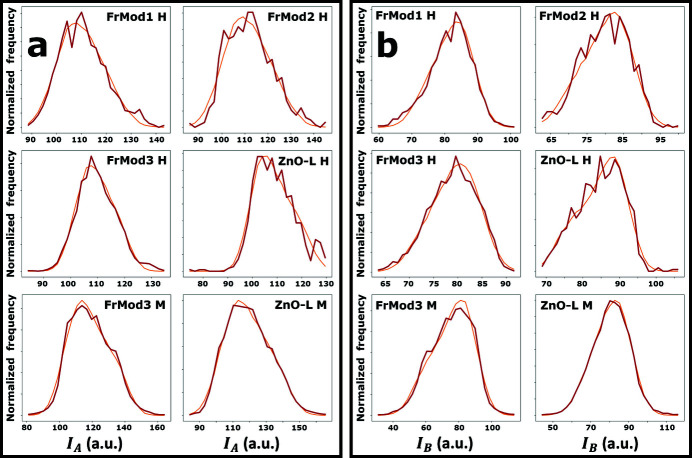
Experimental distributions (red) of *I*_*A*_ (*a*) and *I*_*B*_ (*b*) fitted according to the alternative dichroic model postulated in equations (12) and (13) (orange).

Fig. 8 ▸ shows the fits obtained for each of the experimental distributions, according to the model proposed in equations (12) and (13) and taking 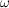 = 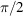 . Here, it can be seen that the skews in the distributions of *I*_*B*_ are now modeled more accurately than they were using the simpler model (see Fig. 6 ▸). The corresponding normalized dichroic model parameters and estimated experimental errors are listed in Tables 1 ▸ and 2 ▸. The uncertainty in each parameter value is calculated from the previously discussed uncertainties *u*_*P*,mod_ and *u*_*P*,exp_ according to

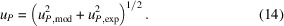




## 4. Discussion   

From the experimental distributions shown in Fig. 4 ▸, it could be observed visually that the distributions obtained from hyperspectral STXM energy stacks are narrower than those obtained from two-energy STXM stacks. Moreover, a difference could be observed between the distributions of *I*_*B*_ obtained from the two-energy STXM stack recorded on the ZnO-L and FrMod3 samples [see Fig. 4 ▸(*d*)]. Having modeled these distributions following the XNLD model proposed in equations (12) and (13), it is now possible to interpret these differences in terms of the dichroic parameters and check their significance based on a statistical hypothesis test. Here, the significance of differences in dichroic parameters was tested using zeta-scores (Analytical Methods Committee, 2016 ▸),

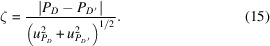

A zeta-score measures the difference between two parameters *P* of data sets *D* and *D*′ normalized to the expected standard deviation of this difference under the assumption that *P*_*D*_ and *P*_*D*′_ are both measures of the same quantity. For instance, a zeta-score of 1.96 indicates that the difference between *P*_*D*_ and *P*_*D*′_ is equal to two standard deviations, meaning that larger differences are only expected in 5% of cases (*p* = 0.05).

Tables 3 ▸ and 4 ▸ show the *p*-values calculated for comparisons of 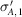 , σ_*A*,2_, σ_*B*,0_, and 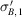 , based on the values listed in Tables 1 ▸ and 2 ▸. The other parameters were left out either because they function as normalization factors (σ_*A*,0_), their difference is not relevant ( 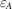 and 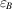 ) or because no statistically significant differences could be found (σ_*B*,2_). It is clear from the tabulated *p*-values that the distributions obtained from the two-energy STXM energy stacks (FrMod3 M and ZnO-L M) exhibit XNLD behavior that is significantly different from the distributions obtained from the hyperspectral STXM energy stacks (FrMod1 H, FrMod2 H, FrMod3 H, and ZnO-L H). These differences are most pronounced in terms of the 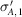 parameter (see Table 3 ▸, bottom two rows), which means that significant differences exist in the amplitude of the dichroic effect of the major transition at 1031 eV, depending on whether data are retrieved using a high or low radiation dose. Comparing the same data sets, less pronounced statistical differences can also be observed in terms of the 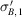 parameter (see Table 4 ▸, final two columns), which measures the amplitude of the dichroic effect of the minor transitions at 1033 eV.

Taken altogether, this provides strong evidence that the XNLD behavior of ZnO is affected by high X-ray radiation doses. To be precise, the amplitude of the XNLD effect was found to be significantly lower after excessive exposure of the sample to a tightly focused synchrotron-generated X-ray beam. As X-ray linear dichroism in ZnO is directly related to the symmetry of its crystal structure, it is hypothesized that the decreased XNLD amplitude is caused by a decrease in the crystallinity of the irradiated crystallites. That is, high X-ray radiation doses are thought to induce a certain level of amorphism which partially breaks the alignment of low-symmetry electronic orbitals. Interestingly, these observations are in direct opposition with the findings of Wang *et al.* who show that exposing ZnO nanowires to soft X-rays (O *K*-edge, ∼550 eV) for several minutes increases the intensity of X-ray induced optical band gap emission with respect to defect-related trap state emission (Wang *et al.*, 2014 ▸). The authors ascribe this observation to a form of radiation-induced annealing — in which the energy deposited by the X-rays enables the crystal to cross energy barriers towards energetically more favorable (more crystalline) configurations. It is likely that in the experiments by Wang *et al.* the radiation dose deposition rate was orders of magnitude lower, producing a distinctly different effect on the material properties of ZnO than when the dose deposition rate is as high as in the STXM experiments described here.

Focusing on the differences between samples, rather than between measurement methods, two *p*-values do raise some suspicion with regards to the dissimilarity of the ZnO-L sample with respect to the three FrMod samples. First, a significant difference (*p* = 5.1 × 10^−3^) in the σ_*B*,0_ parameter is demonstrated for the distributions obtained on the FrMod3 and ZnO-L samples from two-energy STXM energy stacks. Second, the comparison of the 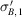 parameter for distributions obtained on the FrMod3 and ZnO-L samples from hyperspectral STXM data sets yields a *p*-value of 0.057. However, since the σ_*B*,0_ parameter does not measure the amplitude of a dichroic effect and the difference in values of 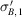 is not formally seen as sufficient evidence of dissimilarity, these results are only regarded as pointing in the direction of a possible relation between the production process of ZnO and its XNLD properties.

This ambiguity in establishing statistically significant dissimilarity between the ZnO-L and FrMod points to a major point of potential improvement in the XLD quantification method described here. With the raw data shown in Fig. 4 ▸ and the model parameter uncertainties listed in Tables 1 ▸ and 2 ▸, it is clear that modeling efforts are strongly affected by the low number of analyzed ZnO crystallites. That is, the fitting of experimental data with model distributions is expected to benefit from including as many crystallite orientations as possible. It is therefore postulated here that the discriminatory power of this XLD quantification method can be significantly improved by analyzing larger (or more) crystallite agglomerates per sample.

## 5. Conclusion   

It has been shown that it is possible to extract information on the X-ray linear dichroism properties of powders by performing STXM with a nanofocused, linearly polarized X-ray beam. A procedure is proposed that involves (1) the identification of symmetrically distinct diagnostic transitions by means of spectral endmember selection, (2) the modeling of the intensity distributions of the diagnostic transitions, and (3) the estimation of uncertainties in the dichroic model parameters by means of a Monte Carlo simulation. This procedure was demonstrated by analyzing STXM data sets obtained on several differently produced ZnO powders in both a hyperspectral and multispectral manner. It was found that the intensity distributions at two diagnostic energies could be modeled most accurately by making use of a model that assumes some degree of spectral overlap. Comparing the estimates of the dichroic model parameters for all data sets revealed that samples that received a lower radiation dose (multispectral method) exhibit a more pronounced XNLD effect than those that received a higher radiation dose (hyperspectral method). Moreover, statistical comparison of the XNLD parameters of a lab-synthesized ZnO sample compared with a commercially produced zinc white point to a possible relationship between the production process of ZnO and its dichroic properties.

## Figures and Tables

**Table 1:** Dichroic model parameters, estimated experimental errors, and corresponding uncertainties for the four hyperspectral STXM data sets; all values are normalized such that σ_*A*,0_ was set to 100 for each data set

FrMod1	Value	*u*_*P*_	FrMod2	Value	*u*_*P*_	FrMod3	Value	*u*_*P*_	ZnO-L	Value	*u*_*P*_
σ_*A*,0_	100	2.2	σ_*A*,0_	100	2.1	σ_*A*,0_	100	1.7	σ_*A*,0_	100	1.8
\sigma _{A,\,1}^{\,\prime}	24.7	5	\sigma _{A,\,1}^{\,\prime}	27.2	4	\sigma _{A,\,1}^{\,\prime}	21.4	3	\sigma _{A,\,1}^{\,\prime}	23.5	2.8
σ_*A*,2_	5.7	4	σ_*A*,2_	7.1	4	σ_*A*,2_	7.5	4	σ_*A*,2_	3.0	3
{\varepsilon }_{A}	4.9	1.0	{\varepsilon }_{A}	5.3	0.9	{\varepsilon }_{A}	3.0	0.6	{\varepsilon }_{A}	3.60	0.8
σ_*B*,0_	71.7	1.9	σ_*B*,0_	68.4	1.9	σ_*B*,0_	71.1	1.8	σ_*B*,0_	72.3	3
\sigma _{B,\,1}^{\,\prime}	16.1	3	\sigma _{B,\,1}^{\,\prime}	17.0	4	\sigma _{B,\,1}^{\,\prime}	10.9	2.9	\sigma _{B,\,1}^{\,\prime}	20.3	4
σ_*B*,2_	14.5	4	σ_*B*,2_	17.6	5	σ_*B*,2_	13.4	4	σ_*B*,2_	16.8	3
{\varepsilon }_{B}	3.24	0.7	{\varepsilon }_{B}	3.4	0.8	{\varepsilon }_{B}	2.7	0.5	{\varepsilon }_{B}	2.6	0.7

**Table 2:** Dichroic model parameters, estimated experimental errors, and corresponding uncertainties for the two multispectral STXM data sets; all values were normalized such that σ_*A*,0_ was set to 100 for both data sets

FrMod3	Value	*u*_*P*_	ZnO-L	Value	*u*_*P*_
σ_*A*,0_	100	2.5	σ_*A*,0_	100	2.6
\sigma _{A,\,1}^{\,\prime}	41.8	5	\sigma _{A,\,1}^{\,\prime}	42.8	4
σ_*A*,2_	14.0	4	σ_*A*,2_	14.2	4
{\varepsilon }_{A}	4.8	0.9	{\varepsilon }_{A}	5.7	0.8
σ_*B*,0_	52.8	4	σ_*B*,0_	65.1	2.0
\sigma _{B,\,1}^{\,\prime}	38	10	\sigma _{B,\,1}^{\,\prime}	21.6	4
σ_*B*,2_	31	9	σ_*B*,2_	24.2	6
{\varepsilon }_{B}	5.6	1.1	{\varepsilon }_{B}	5.49	0.5

**Table 3:** Statistical comparison of the dichroic model parameters \sigma_{A,1}^{\,\prime} (left of the diagonal) and σ_*A*,2_ (right of the diagonal) in terms of *p*-values; all *p*-values lower than 0.05 are highlighted in bold

	FrMod1 H	FrMod2 H	FrMod3 H	ZnO-L H	FrMod3 M	ZnO-L M
FrMod1 H	–	0.80	0.75	0.59	0.13	0.13
FrMod2 H	0.70	–	0.94	0.41	0.23	0.19
FrMod3 H	0.57	0.23	–	0.37	0.27	0.23
ZnO-L H	0.83	0.42	0.61	–	**0.028**	**0.028**
FrMod3 M	**0.016**	**0.028**	**4.7 × 10^−4^**	**1.4 × 10^−3^**	–	0.97
ZnO-L M	**5.1 × 10^−3^**	**6.9 × 10^−3^**	**1.7 × 10^−5^**	**6.3 × 10^−5^**	0.87	–

**Table 4:** Statistical comparison of the dichroic model parameters σ_*B*,0_ (left of the diagonal) and \sigma _{B,1}^{\,\prime} (right of the diagonal) in terms of *p*-values; all *p*-values lower than 0.05 are highlighted in bold, while *p*-values lower than 0.10 but higher than 0.05 are highlighted in italic

	FrMod1 H	FrMod2 H	FrMod3 H	ZnO-L H	FrMod3 M	ZnO-L M
FrMod1 H	–	0.86	0.23	0.40	**0.036**	0.27
FrMod2 H	0.23	–	0.23	0.56	*0.057*	0.42
FrMod3 H	0.82	0.32	–	*0.057*	**9.3 × 10^−3^**	**0.028**
ZnO-L H	0.87	0.27	0.73	–	0.11	0.82
FrMod3 M	**1.7 × 10^−5^**	**4.7 × 10^−4^**	**2.6 × 10^−5^**	**9.6 × 10^−5^**	–	0.13
ZnO-L M	**0.016**	0.23	**0.028**	**0.046**	**5.1 × 10^−3^**	–
